# Diabetic neuropathy’s immune-metabolic network: mechanistic complexity, therapeutic challenges, and the path forward

**DOI:** 10.3389/fimmu.2026.1804076

**Published:** 2026-04-23

**Authors:** Wenjuan Sun, Kunpeng Yang, Yangyang Song

**Affiliations:** 1 Jilin Provincial People’s Hospital, Changchun, China; 2 Changchun University of Chinese Medicine, Changchun, China; 3 Department of Thoracic Oncology Surgery II, Jilin Cancer Hospital, Changchun, Jilin, China

**Keywords:** diabetic neuropathy, immune-metabolic network, metabolic reprogramming, PANoptosome, Schwann cells

## Abstract

Diabetic neuropathy (DN) is the most prevalent and debilitating complication of diabetes, with a notable absence of effective disease-modifying therapies in clinical practice. This article proposes a shift in the pathological progression of DN from focusing on “metabolic toxicity” to an integrated dysfunction within the “immune-metabolic network.” We analyze the core mechanisms underlying this network and highlight how the diabetic microenvironment may drive immune cells to shift abnormally from the “Warburg effect” to “metabolic inflexibility” and “metabolic paralysis,” ultimately failing to resolve inflammation and causing persistent tissue damage. Furthermore, we identify a “zero-sum game” between Schwann cells (SCs) in their roles in immune response and metabolic support. Pro-inflammatory signals trigger the collapse of the “lactate shuttle” mechanism, exposing neurons to the dual insults of “hunger” and “toxicity.” At the molecular level, we highlight ZBP1 as a critical switch, sensing mitochondrial damage and is proposed to trigger the assembly of the PANoptosome complex, which forms the terminal execution pathway for neurodegenerative lesions. Given the current gap between animal models and clinical realities, we propose employing spatial transcriptomics to examine subpopulation differences between the nerve sheath and endoneurium, alongside the development of novel precision therapies targeting NLRP3 or utilizing metabolic reprogramming to restore immune repair functions. In conclusion, framing DN within the immune-metabolic network provides a new approach to overcoming the therapeutic impasse and developing truly effective interventions.

## 1 Introduction

Diabetic neuropathy (DN) is the most common chronic complication of diabetes, affecting nearly 50% of the global diabetic population, which totals hundreds of millions (1, 2). Its clinical manifestations are diverse, with distal symmetric polyneuropathy (DSPN) being the most common form. The primary features include sensory loss, chronic pain, and motor dysfunction. In severe cases, this can lead to foot ulcers, infections, and even amputations, significantly impairing patients’ quality of life and increasing mortality risk (3, 4). Although strict blood glucose control effectively delays DN progression in type 1 diabetes, its effect in the more prevalent type 2 diabetes is relatively limited (3). Currently, clinical treatment is primarily symptomatic, involving the use of anticonvulsants and antidepressants to alleviate neuropathic pain (5). However, no disease-modifying therapy (DMT) is currently available that can effectively halt or reverse nerve damage (1). This significant unmet clinical need calls for a deeper understanding of the pathogenesis of DN to identify novel therapeutic targets.

A historical review of DN’s pathological mechanisms reveals a process of increasing depth and complexity. Early studies concentrated on two core mechanisms: metabolic toxicity induced by hyperglycemia and neuroischemia and hypoxia resulting from diabetes-related microvascular damage (6). As research progressed, a more comprehensive “multiple injury” hypothesis gradually emerged. This hypothesis integrated several interrelated pathological processes, such as activation of the polyol pathway, formation of advanced glycation end products (AGEs), activation of protein kinase C (PKC), oxidative stress, mitochondrial dysfunction, and a lack of neurotrophic factors (7). However, it remains a subject of ongoing debate whether these traditional neuron- and vasculature-centered models can fully explain all pathological features of DN, particularly its persistent, low-grade chronic inflammation (8). Recent evidence has increasingly shown that neuroinflammation is not merely a secondary response to nerve damage, but rather a central driver of DN’s onset and progression (8, 9). At the same time, breakthroughs in immunology have revealed that the functional state of immune cells is closely tied to their internal metabolic patterns, a concept known as “immune-metabolism” (10, 11). This new theory provides a novel framework for understanding chronic inflammation in DN: systemic metabolic disturbances in diabetes not only directly damage neurons, but more importantly, reshape the metabolism and function of immune cells, making them prone to a pro-inflammatory phenotype and thereby initiating and sustaining a persistent “immune battle” within the peripheral nervous system.

Consequently, understanding DN’s pathological mechanisms is undergoing a paradigm shift, transitioning from a fragmented view of metabolic or immune factors to an integrated “immune-metabolic-neuro” interactive network model (12). To address current knowledge gaps and build upon this paradigm shift, this perspective proposes the central hypothesis that in the pathogenesis of DN, a self-amplifying vicious cycle exists between systemic metabolic dysregulation and immune system dysfunction, acting as the central engine driving the ongoing progression of nerve damage. Specifically, metabolic stressors such as hyperglycemia and lipotoxicity serve as initiating factors that not only directly damage neurons and Schwann cells(SCs) but, more critically, activate both innate and adaptive immune responses. Activated immune cells (e.g., macrophages, T cells) undergo metabolic reprogramming, reinforcing their pro-inflammatory state and releasing numerous inflammatory mediators, further exacerbating local tissue metabolic dysregulation and oxidative stress. This process damages the blood-nerve barrier, ultimately resulting in axonal degeneration and neuronal death. Immune-metabolic dysregulation-driven programmed cell death, such as PANoptosis, may serve as a crucial link connecting upstream metabolic disturbances with downstream nerve damage (12). Therefore, breaking this vicious cycle—especially by targeting key regulatory nodes within the immune-metabolic network—could offer unprecedented opportunities for developing disease-modifying therapies for DN.

## 2 Key components analysis: critical dialogues within the immune-metabolic network

The immune-metabolic network in DN is a complex system made up of various molecules and cell types. Understanding its core components and their interactions is essential to uncovering the full pathophysiological picture of DN. Several metabolic abnormalities in the diabetic environment are hypothesized to act as “first messengers,” initiating and amplifying immune responses. While definitive *in vivo* causality in human DN remains an area of active investigation, these mechanisms are strongly inferred from related systemic metabolic disorders and established inflammatory models.

Chronic hyperglycemia first leads to the accumulation of AGEs in tissues through non-enzymatic glycation reactions. These AGEs bind to their receptor, RAGE, activating downstream signaling pathways like NF-κB. This triggers the release of various pro-inflammatory cytokines (e.g., TNF-α, IL-1β) by macrophages, microglia, and endothelial cells, creating a crucial molecular link between metabolic dysregulation and inflammation (13).

Lipid metabolism abnormalities, commonly associated with type 2 diabetes, secondly lead to increased levels of free fatty acids (e.g., palmitic acid) and sphingolipids (e.g., ceramides) in circulation. These molecules act as damage-associated molecular patterns (DAMPs), directly activating Toll-like receptor 4 (TLR4) and the NLRP3 inflammasome, which in turn triggers robust inflammatory responses (1, 14).

Insulin resistance also plays a significant role in immune cell dysfunction. When insulin signaling is impaired, energy metabolism and functional differentiation in immune cells, such as macrophages and T cells, are disrupted. This makes them more prone to a pro-inflammatory phenotype, exacerbating both systemic and local inflammatory cascades (15). Metabolic signals eventually activate various immune cells, transforming systemic dysregulation into localized nerve tissue damage (16).

As the core of the innate immune system, tissue-resident and infiltrating peripheral macrophages play a highly active and detrimental role in DN. In the early stages of the disease, resident macrophages (and microglia in the spinal cord) may exert a protective role by phagocytosing cellular debris and promoting tissue repair (17). However, under sustained metabolic stress, such as in high-fat diet-induced metabolic syndrome or diabetes, there is a profound accumulation of pro-inflammatory (M1) macrophages within peripheral nerves, including the sciatic nerve (18). This macrophage infiltration is not a passive consequence but a primary driver of long-lasting neuroinflammation that directly precipitates neuropathic pain behaviors like mechanical allodynia (18). The pathogenic impact of these macrophages extends deep into nerve physiology. For instance, the activation of the receptor for advanced glycation end products (RAGE) in infiltrating macrophages induces severe local insulin resistance, which subsequently causes dorsal root ganglion (DRG) neuron atrophy and slows retrograde axonal transport (RAT) (19). Furthermore, this macrophage-driven inflammation creates a severe cytokine dysregulation within the DRG and peripheral skin—characterized by the upregulation of pro-inflammatory cytokines (TNF-α, IL-1β, IL-6) and the suppression of anti-inflammatory IL-10—which recruits additional inflammatory cells (e.g., Langerhans cells) to nerve terminals, cementing a state of chronic, unresolved pain (20).

Notably, the metabolic overload underlying this macrophage polarization presents a unique “paradox” in the diabetic context. Unlike the “Warburg effect” (compensatory enhancement of glycolysis) that typically accompanies M1 polarization in classic immunological paradigms, recent evidence suggests that diabetic macrophages experience profound “metabolic inflexibility” (21). Under the influence of sustained insulin resistance, the impairment of the PI3K-Akt-mTOR signaling pathway leads to a downregulation of glucose transporter 1 (GLUT1); consequently, both glycolytic flux and oxidative phosphorylation are simultaneously inhibited (21).

This transition from the adaptive “Warburg effect” (upregulated glycolysis) to a state of “metabolic paralysis” is a defining feature of chronic DN. Unlike acute inflammatory models where increased glycolytic flux powers M1 macrophages to clear pathogens, the sustained insulin resistance in diabetic nerves impairs the cellular bioenergetic capacity (18). Consequently, macrophages lack the ATP required to initiate the energy-intensive phenotypic switch to M2 repair-type cells, trapping the nerve microenvironment in a “smoldering” inflammatory state (19).

Critique & Limitations: However, several critical questions remain regarding this “metabolic paralysis” model. First, most current evidence stems from rodent models such as the db/db mouse or HFD-induced T2DM; whether human diabetic macrophages exhibit an identical bioenergetic collapse in long-term clinical settings remains to be confirmed through high-resolution metabolic flux analysis in nerve biopsies. Second, it is still debated whether this paralysis is a primary driver of nerve damage or a secondary consequence of chronic mitochondrial DNA (mtDNA) leakage from neighboring axons. Addressing these gaps is essential for determining if metabolic reprogramming of macrophages can serve as a viable disease-modifying strategy.

Mechanistically, lacking sufficient ATP to drive energy-consuming phagocytic clearance and the phenotypic switch to the M2 repair type, local neuroinflammation falls into a “smoldering” state—neither fully erupting nor resolving. This “resolution failure” is a core mechanism driving the chronicity of DN and the refractory nature of neural tissue healing. Furthermore, the adaptive immune system is also deeply involved in this prolonged pathogenesis. Studies have found that the infiltration of pro-inflammatory T cell subsets, such as Th1 and Th17, along with their secretion of cytokines like IFN-γ and IL-17, further amplifies the inflammatory cascade (22, 23). Concurrently, the impaired number or function of regulatory T cells (Tregs) leads to a severe imbalance in immune homeostasis (24).

Additionally, SCs, the primary glial cells of the peripheral nervous system, undergo a role shift in the diabetic environment. This shift is evident not only in their secretion of inflammatory factors but also in the fundamental disruption of their metabolic support function (25). Under normal conditions, SCs act as an essential “metabolic fueling station” for axons: they take up glucose and perform aerobic glycolysis to produce lactate, which is then transported to axons via monocarboxylate transporters (MCT1/2), serving as a vital energy source for axonal mitochondria to produce ATP (26). However, in the diabetic environment, SCs transform into a “quasi-immune cell” state, expressing MHC-II molecules and secreting chemokines to recruit immune cell infiltration (27).

This functional shift is based on a redistribution of resources between immunity and metabolism, where SCs redirect limited cellular resources from metabolic support to immune defense (28). Studies have shown that pro-inflammatory signals (e.g., TNF-α) and sustained hyperglycemia induce SCs to downregulate key metabolic enzymes (e.g., PKM2) and transporters like MCT1, resulting in a profound impairment of the “lactate shuttle” mechanism (29, 30). This transformation has disastrous consequences: SCs shift from being “supporters” of neurons to “contributors” to inflammation (31, 32).

This functional shift represents a “bioenergetic zero-sum game” where SCs divert limited ATP from axonal metabolic support to active immune defense (27, 29). Recent evidence suggests that mitochondrial dysfunction within SCs acts as the primary “molecular trigger” for this network collapse. Damaged mitochondria release reactive oxygen species (ROS) and mtDNA into the endoneurial space, which serve as potent DAMPs that recruit and activate peripheral macrophages (19). This creates a vicious cycle: mitochondrial failure drives immune infiltration, which in turn exacerbates oxidative stress and further impairs the “lactate shuttle,” leading to an energy crisis in the axons they support (20).

Critique & Limitations: Despite its mechanistic elegance, this “lactate shuttle” model faces the challenge of “spatial heterogeneity.” It remains debated whether SC metabolic failure occurs uniformly across the entire nerve length or is localized to distal terminals where glucose fluctuations and oxidative stress are most extreme. Furthermore, while rodent models demonstrate a clear link between SC glycolysis and axonal health, translating these findings to humans is complicated by the significantly greater length of human axons, which may place even higher metabolic demands on SCs than currently captured in experimental settings.

Consequently, axons endure immune attacks while simultaneously being deprived of their optimal energy supply. It is important to note that current *in vivo* data primarily indicate reduced transporter function and metabolic deficits, rather than a full loss of Schwann cell support. This “double blow of starvation and toxicity” is a profound metabolic-immune mechanism that drives the progressive degeneration of axons in DN.

Within the immune-metabolic network, specific signaling molecules and pathways serve as hubs for information integration and amplification. Classic pro-inflammatory factors, such as TNF-α, IL-1β, and IL-6, act as key effector molecules in the neuroinflammatory microenvironment of DN. These factors can directly induce neuronal apoptosis, inhibit axonal regeneration, and further activate immune cells through positive feedback loops, a mechanism also observed in intestinal inflammation (8, 33). The NLRP3 inflammasome, an intracellular multiprotein complex, senses various metabolic stress signals, cleaves and activates caspase-1, and promotes the release of IL-1β and IL-18, acting as a key “amplifier” of pyroptosis and neuroinflammation (34, 35). Additionally, pattern recognition receptors such as TLR2 and TLR4 can be activated by endogenous DAMPs, triggering signaling pathways that activate NF-κB and promote the transcription of inflammatory genes (36).

The body also has endogenous systems to counteract immune-metabolic imbalance, but these systems are often insufficient under prolonged diabetic conditions. In addition to neurotrophic factors with anti-inflammatory and pro-survival functions (e.g., MANF), intracellular energy sensors such as AMPK and Sirtuins (e.g., SIRT1) play a crucial role in suppressing inflammation. In the context of diabetes, these systems are typically suppressed, leading to a decline in metabolic homeostasis and reduced anti-inflammatory efficacy (14). Furthermore, microRNAs (e.g., miR-146a, miR-155) finely regulate immune-metabolic pathways in DN by targeting mRNA, thus influencing the intensity and duration of the inflammatory response (35).

Crucially, the immune-metabolic dysregulation described above directly impacts neuronal excitability, a central and well-established driver of DN pathophysiology (1, 37). Pro-inflammatory cytokines (e.g., TNF-α, IL-1β) within the local microenvironment not only sustain inflammation but directly modulate the transcription and post-translational modification of critical ion channels (38, 39). For instance, voltage-gated sodium channels (Nav), particularly Nav1.7 and Nav1.8, are significantly upregulated and sensitized in sensory neurons (40). This alteration lowers the neuronal excitation threshold and drives ectopic firing, serving as the cellular basis for neuropathic pain (40). Conversely, voltage-gated potassium channels (Kv), which normally act as excitability “brakes,” are widely downregulated, thereby prolonging action potentials and exacerbating the hyperexcitable state (40, 41).

Furthermore, this functional hyperexcitability is intimately linked to structural degeneration through a profound energy crisis (37). Action potential generation and the subsequent restoration of ionic homeostasis via Na+/K+-ATPases and Ca2+-ATPases are highly energy-demanding processes (1). The aforementioned impairment of the Schwann cell “lactate shuttle” creates a severe bioenergetic deficit. When axons are deprived of optimal metabolic support from SCs, the immense energetic cost of maintaining altered ion channel activity under oxidative stress becomes completely unsustainable (37). This mismatch between skyrocketing energy demand and collapsing metabolic supply forces mitochondria into a state of failure, acting as a crucial mechanistic bridge that translates functional neuronal hyperexcitability into structural sensory fiber degeneration (1, 42).

## 3 Mechanism integration: from systemic dysregulation to cascading local nerve injury

The development of DN is a complex, multi-stage, and multi-level pathological process (43), with the immune-metabolic network continuously driving this progression (44–46). To better understand this intricate mechanism, we can frame it within a cascading reaction model that outlines the gradual progression from systemic metabolic dysregulation to localized nerve damage.

In the early stages of diabetes, the body is in a prolonged state of systemic hyperglycemia, hyperlipidemia, and insulin resistance (47, 48). These metabolic abnormalities result in the accumulation of AGEs, oxidized low-density lipoprotein (ox-LDL), and various cytokines, such as TNF-α and IL-1β, in the circulation. Together, these molecules push circulating immune cells, such as monocytes and lymphocytes, into a “pre-activated” or “sensitized” state (49, 50). This means that their reactivity to subsequent stimuli is heightened, and their activation threshold is significantly lowered (50). This sets the stage for the subsequent inflammatory cascade (51).

The peripheral nervous system is largely shielded from systemic inflammation by the blood-nerve barrier (BNB) (52, 53). The BNB is composed of tightly connected microvascular endothelial cells, pericytes, and the basement membrane (54). However, under sustained metabolic stress and systemic low-grade inflammation, BNB endothelial cell function is compromised, disrupting tight junctions between cells and increasing barrier permeability (55). This creates a “gap,” allowing pre-activated immune cells to cross the barrier and infiltrate the nerve endoneurium, which is usually considered an immune-privileged region (38). Consequently, systemic inflammation is introduced into localized nerve tissue.

Once infiltrating the nerve endoneurium, immune cells undergo significant and complex “metabolic reprogramming,” which underpins their transition from a patrol state to an effector/pathogenic state (56). While classic M1 polarization is typically linked to a shift toward aerobic glycolysis (i.e., the “Warburg effect”), the situation in the context of diabetes, a chronic pathological environment, is much more complex than this simplified model suggests. Recent research suggests that, under sustained metabolic stress and impaired key insulin signaling pathways, diabetic macrophages may experience “metabolic inflexibility” (21).

In this state, macrophages not only fail to effectively initiate oxidative phosphorylation, but their glycolytic flux may also be significantly inhibited, presenting characteristics of “metabolic paralysis” or “energy failure” (57). This collapse of bioenergetics has dual pathogenic significance: on one hand, it reinforces the cell’s pro-inflammatory phenotype; on the other hand, due to insufficient energy for energy-intensive processes like phenotype switching and phagocytic clearance, it leads to failure in resolving inflammation. As a result, the nerve tissue remains in a chronic, unresolved “smoldering” inflammatory state, preventing healing (6, 58). This activated immune cell response, combined with damaged SCs, neurons, and endothelial cells, creates a self-sustaining and continuously amplifying chronic inflammatory microenvironment.

This harsh, chronic inflammatory microenvironment exposes neurons and SCs to the dual blows of metabolic toxicity and immune attack. First, mitochondrial dysfunction and oxidative stress are greatly amplified, leading to insufficient cellular energy and triggering energy failure (37). Second, axonal transport is disrupted, resulting in distal axonal degeneration. This persistent stress state eventually initiates a controlled programmed cell death process—PANoptosis (12).

Unlike single modes of cell death, PANoptosis is a coordinated process involving apoptosis, pyroptosis, and necroptosis, regulated by the PANoptosome complex. The pathological microenvironment of DN provides the ideal triggering signals for its assembly (12). Specifically, the classic pro-inflammatory factor TNF-α from M1 macrophages can activate extrinsic apoptosis (Caspase-8) and necroptosis (RIPK1/3) pathways. Meanwhile, hyperglycemia-driven oxidative stress and ROS generated from mitochondrial damage further activate the NLRP3 inflammasome, initiating the pyroptosis pathway (Caspase-1) (12).

Crucially, Z-DNA binding protein 1 (ZBP1) is proposed to act as the central molecular switch connecting metabolic dysregulation and immune response in this network (12). However, it must be carefully acknowledged that there is currently no direct *in vivo* evidence establishing ZBP1-dependent PANoptosis as a dominant mechanism in this specific neuropathic context. As most existing ZBP1 data are derived from viral, interferon, and systemic inflammatory models, its precise regulatory role in DN remains an exploratory but highly promising frontier. Under diabetic stress, mitochondrial dysfunction in neurons and SCs causes the leakage of mtDNA into the cytoplasm, where it is recognized as a danger signal by ZBP1. This recognition is hypothesized to trigger and sustains the assembly of the PANoptosome complex (59).

This terminal execution is powered by a catastrophic failure in axonal bioenergetics. Chronic metabolic stress leads to an accumulation of damaged mitochondria that the distal axon, far from the cell body, cannot effectively recycle (1, 59). This bioenergetic crisis does not merely starve the axon; the resulting leakage of mtDNA and ROS into the cytoplasm acts as a potent internal Danger-Associated Molecular Pattern (DAMP), specifically nucleating the assembly of the ZBP1-PANoptosome (12, 59). Activation of this complex triggers a cascade of cell death programs, including pyroptosis and necroptosis, which accelerate the dissolution of the BNB and recruit further waves of sensitized macrophages (32, 60).

**Figure 1:**
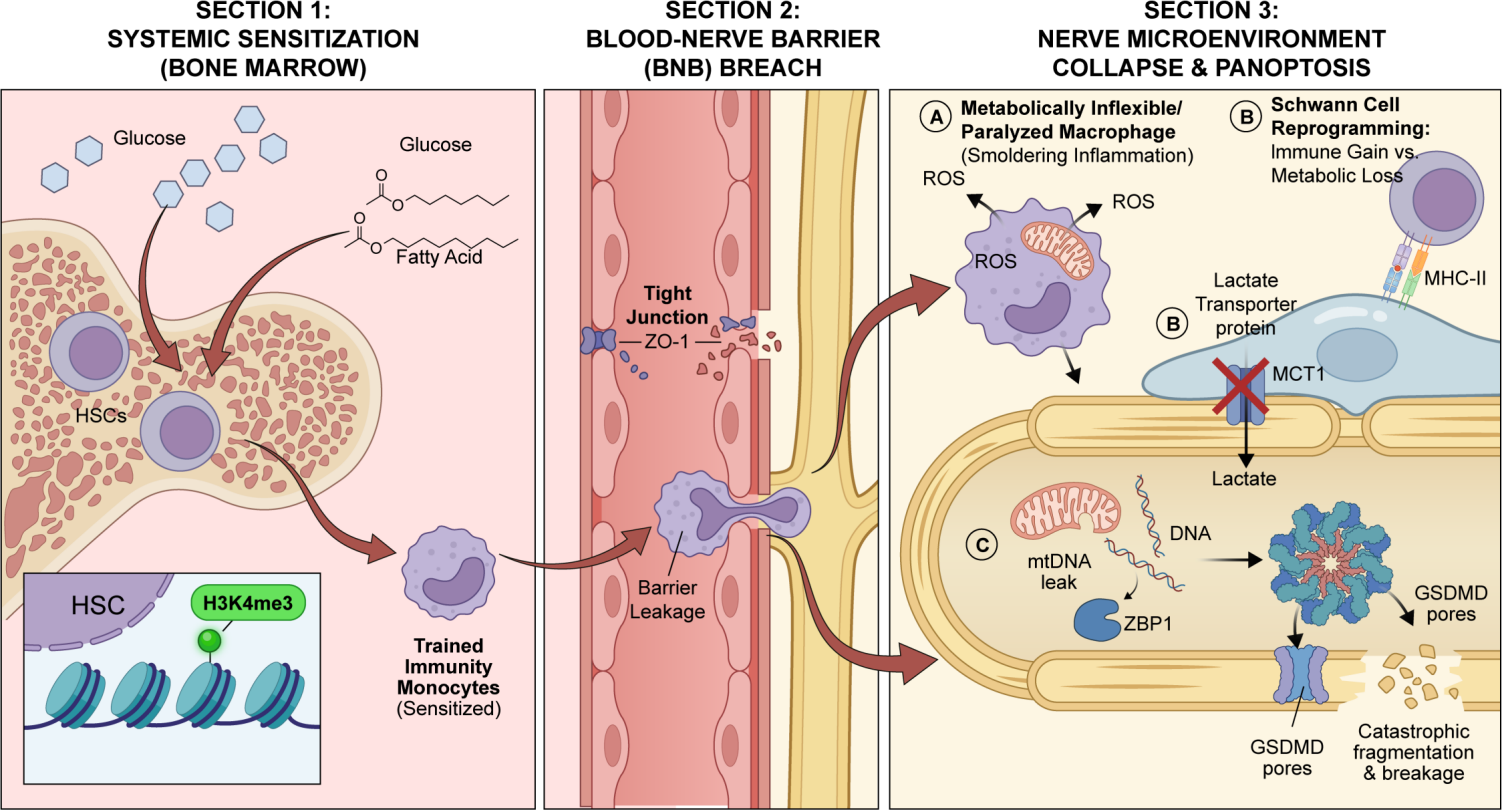
From systemic sensitization to local neurovascular collapse: a spatiotemporal mechanism panorama. This figure illustrates the progressive development of diabetic neuropathy pathology across three distinct anatomical locations. Section 1: Systemic Sensitization (Bone Marrow). High circulating levels of glucose and fatty acids in the diabetic state act on Hematopoietic Stem Cells (HSCs) within the bone marrow. This exposure induces epigenetic reprogramming in HSCs, specifically shown as H3K4me3 histone methylation. This process creates “trained immunity,” resulting in the generation of sensitized monocytes that are primed for inflammation before they enter peripheral tissues. Section 2: Blood-Nerve Barrier (BNB) Breach. Chronic hyperglycemia leads to the degradation of tight junction proteins, such as ZO-1, between endothelial cells, compromising the integrity of the blood-nerve barrier. This barrier leakage allows the trained, sensitized monocytes from the circulation to infiltrate the nerve endoneurium. Section 3: Nerve Microenvironment Collapse & PANoptosis. Within the nerve microenvironment, several critical pathological events occur: **(A)** Macrophage Metabolic Paralysis: Infiltrated macrophages, under conditions of insulin resistance and lipotoxicity, do not exhibit a classic Warburg effect but rather “metabolic inflexibility”. Their mitochondria are damaged and swollen, leading to the production of reactive oxygen species (ROS). This metabolic failure prevents the resolution of inflammation, resulting in a chronic, smoldering inflammatory state. **(B)** The Schwann Cell Trade-off: In response to inflammatory stress, Schwann cells undergo reprogramming. They downregulate the lactate transporter MCT1, thereby ceasing the essential lactate shuttle that provides metabolic support to axons. Simultaneously, they upregulate MHC-II to present antigens to T-cells, representing a critical trade-off between immune function and metabolic support. **(C)** Axonal PANoptosis: The combined effects of energy deprivation from Schwann cells and inflammatory attack trigger axonal cell death. The innate immune sensor ZBP1 detects mitochondrial DNA (mtDNA) that has leaked from damaged axonal mitochondria. This detection initiates the assembly of the PANoptosome complex, which executes cell death through pore-forming proteins like GSDMD, leading to rapid axonal fragmentation and breakage.

Consequently, the activation of this molecular switch not only exacerbates the inflammatory collapse within nerve tissue but also leads to the complete loss of nerve fiber integrity through cell membrane perforation (mediated by GSDMD) and cell lysis. This cascading response is amplified by several positive feedback loops, ultimately causing progressive axonal degeneration and demyelination, which trigger the classic clinical symptoms of DN. This represents the terminal execution mechanism driving neurodegenerative lesions (55). This multi-stage transition from systemic immune sensitization to the final execution of axonal PANoptosis is systematically integrated in **Figure 1**.

Critique & Limitations: Despite the compelling nature of the ZBP1-PANoptosome model, its dominance in the human diabetic context remains to be definitively established. Currently, the most robust *in vivo* evidence for ZBP1-mediated cell death is derived from acute viral infection or high-dose toxicity models, which exhibit far more rapid kinetics than the slow, “smoldering” pathology of human DN (12, 61). Extrapolating these acute findings to a decades-long metabolic disease carries significant translational risks. Furthermore, the inherent redundancy within the PANoptosome—where blocking a single pathway like apoptosis may simply shift the execution to necroptosis—presents a formidable barrier to traditional single-target drug development (62, 63). Future interventions must move upstream to preserve mitochondrial bioenergetic stability rather than merely inhibiting the terminal executioners.

## 4 Current challenges and knowledge gaps

Although the immune-metabolic network provides a robust theoretical framework for understanding the complex pathogenesis of DN, translating this knowledge into effective clinical diagnostic and therapeutic strategies remains challenging.

Currently, most research on DN mechanisms heavily relies on animal models, particularly those induced by streptozotocin (STZ) to create acute hyperglycemia models (64). While these models are valuable for uncovering acute pathological pathways mediated by hyperglycemia, a significant “model gap” exists between these models and the true pathophysiological conditions of human DN, particularly in the context of type 2 diabetes (65).

First, the STZ model is essentially a type 1 diabetes model, simulating absolute insulin deficiency (66), and its immune responses are primarily driven by a single factor—”glucotoxicity.” However, human DN often occurs in the context of long-term, fluctuating hyperglycemia, accompanied by multiple comorbidities in type 2 diabetes, such as obesity, hyperlipidemia, and, most critically, insulin resistance (67). Due to the absence of insulin resistance, a core factor in human disease, the STZ model fails to replicate the “metabolic inflexibility” or “metabolic paralysis” states driven by lipotoxicity in immune cells. This fundamental difference in the model explains why many candidate drugs that show significant anti-inflammatory or antioxidant effects in STZ mice fail in clinical trials, particularly those involving type 2 diabetes patients.

Beyond the STZ model, selecting appropriate animal platforms is critical for replicating the multifaceted immune-metabolic microenvironment of human DN. The db/db mouse, carrying a spontaneous mutation in the leptin receptor (Lepr), is widely utilized due to its reliable development of obesity, severe hyperglycemia, and neuropathic features, such as intraepidermal nerve fiber (IENF) loss by 24 weeks (68, 69). However, its rapid metabolic collapse often bypasses the crucial “prediabetic” phase seen in humans, potentially missing the early windows of macrophage “pre-activation” and metabolic reprogramming (69, 70).

To better simulate the lifestyle-driven evolution of human DN, diet-induced obesity (DIO) models have gained prominence. Notably, the choice of diet composition profoundly shapes the immune-metabolic framework. While a standard High-Fat Diet (HFD) induces a gradual progression of insulin resistance and mild macrophage infiltration, the addition of refined sugars in the High-Fat/High-Sucrose (HFHS) diet (Western diet) triggers a more aggressive, synergistic toxicity (69, 71). As detailed in **Table 1**, the HFHS model leads to a more rapid recruitment of pro-inflammatory cells and a “cliff-like” drop in sensory nerve conduction velocity (NCV), mirroring the terminal neurodegenerative landscape of human DN more closely than standard HFD models (71).

**Table 1:** Metabolic and neuro-immune profiles of HFD vs. HFHS mouse models.

Feature	Standard high-fat diet (HFD)	High-fat/high-sucrose (HFHS)
Systemic Metabolism	Slow-onset obesity and insulin resistance; mild hyperglycemia.	Similar glucose intolerance but significantly accelerated hepatic steatosis.
Neuro-Immune Environment	Gradual accumulation of pro-inflammatory markers and macrophages.	Aggressive inflammatory milieu; rapid and intense macrophage recruitment.
Nerve Conduction (NCV)	Mild-to-moderate slowing of sensory and motor NCV.	"Cliff-like" damage to sensory NCV; motor NCV similar to HFD.
Pain/Sensory Phenotype	Gradual tactile allodynia and delayed thermal hyperalgesia.	Severe allodynia and profound thermal hypoalgesia (sensory loss).
Structural Fiber Loss	Moderate reduction in IENF density.	Near-complete terminal denervation and catastrophic IENF loss.

Therefore, future research should transition from reliance on single models to multi-gene obesity models, humanized organ models (Organ-on-a-chip), or neural organ systems containing human neurons, SCs, and immune cells. Only within platforms that can accurately simulate the complex coupled environment of “metabolic dysfunction-immune dysregulation” can we genuinely identify disease-modifying targets with clinical translational potential.

**Figure 2:**
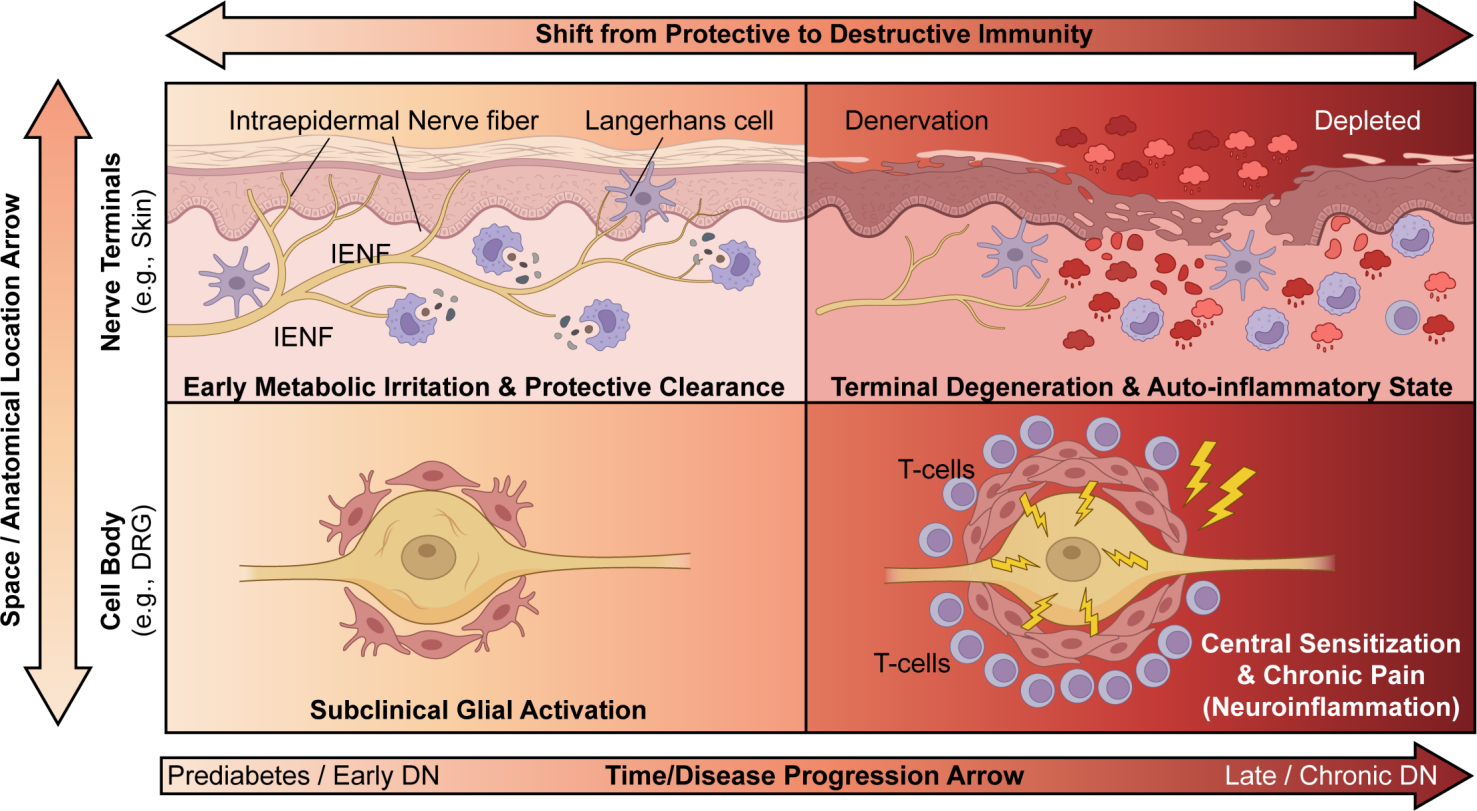
Spatiotemporal heterogeneity of immuno-metabolic responses in diabetic neuropathy (DN). This matrix illustrates that DN pathology is not uniform but varies significantly depending on the disease stage (X-axis: Time/Disease Progression) and anatomical location (Y-axis: Space/Anatomical Location). The overarching arrow at the top indicates the general trend of the immune response shifting from an early protective phase to a late destructive phase as the disease progresses. Top-Left Quadrant: Nerve Terminals (Early DN). In the early stages of prediabetes or DN, metabolic irritation in the skin triggers a mild immune response. Intraepidermal nerve fibers (IENF) remain relatively intact. Resident immune cells, such as Langerhans cells and macrophages, are shown performing a protective surveillance role, engaging in the clearance of cellular debris through phagocytosis. This state is characterized by “Early Metabolic Irritation & Protective Clearance.” Top-Right Quadrant: Nerve Terminals (Late DN). With chronic, prolonged disease, the local environment shifts to a severe “Terminal Degeneration & Auto-inflammatory State”. Protective Langerhans cells are depleted. The tissue exhibits significant denervation with a loss of IENFs. Infiltrating immune cells drive a maladaptive, auto-inflammatory response, releasing cytokines (represented by red clouds) that further damage remaining nerve structures. Bottom-Left Quadrant: Cell Body (Early DN). At the level of the sensory neuron cell body within the Dorsal Root Ganglion (DRG), early changes are subtle. The neuron appears relatively healthy, but surrounding satellite glial cells show signs of “Subclinical Glial Activation,” such as mild hypertrophy, responding to early metabolic stress signals. Bottom-Right Quadrant: Cell Body (Late DN). In late-stage DN, the DRG presents with profound neuroinflammation. Satellite glial cells become hyper-activated and enlarged, forming a dense barrier around the neuron. T-cells infiltrate the ganglia and cluster around the neuron-glial unit. This maladaptive inflammatory environment drives “Central Sensitization & Chronic Pain,” indicated by lightning bolt icons representing spontaneous or amplified pain signaling. Implication: This model highlights the critical need for therapeutic strategies that are tailored to the specific stage and anatomical site of DN pathology, moving beyond a uniform treatment approach.

DN is not a homogeneous disease; its clinical presentation and pathological features display significant spatiotemporal heterogeneity (72). It affects various types of nerve fibers, leading to symptoms such as numbness, reduced sensation, or severe pain (73). A critical structural hallmark of this progression, which directly correlates with the severity of clinical sensory deficits, is the progressive loss of IENF. As conceptualized in **Figure 2**, early DN is characterized by “Early Metabolic Irritation” where IENFs remain relatively intact despite metabolic stress, and resident immune cells exert a protective surveillance role. However, as the disease advances to a late-stage auto-inflammatory state, the depletion of these protective cells and the infiltration of pathogenic immune cells drive extensive terminal denervation, extensive fibrosis, and irreversible structural damage (74, 75), culminating in severe nerve fiber loss. Current “snapshot” studies lack a comprehensive, dynamic depiction of the immune-metabolic network, limiting the development of precise intervention strategies (76). A single biomarker or target may not be applicable to all patients or stages of the disease (77). This necessitates a more granular understanding of the disease’s spatiotemporal evolution, which delineates the distinct immune-metabolic signatures across different anatomical compartments and functional stages.

Immune-metabolic dysregulation is a common feature of many chronic diseases, and ensuring the specificity of these mechanisms remains a scientific challenge. In addition to DN, neurodegenerative diseases such as Alzheimer’s disease, as well as complications like diabetic nephropathy and retinopathy, exhibit similar dysregulation (78).

Therefore, does the immune-metabolic network in DN have specific molecular characteristics? How can we distinguish these specific changes from common inflammatory responses? Answering these questions is crucial for developing highly targeted therapies with minimal side effects. Currently, our understanding of DN-specific immune subsets and metabolic pathways remains insufficient.

Directly targeting the immune-metabolic network also presents therapeutic challenges. Broad-spectrum systemic immunosuppressants, such as corticosteroids, have significant side effects, including increased infection risks and interference with blood glucose control, limiting their clinical application (79). Additionally, the BNB not only prevents immune cells from entering but also restricts the penetration of macromolecules and hydrophilic drugs. Developing strategies that can effectively penetrate the BNB and exert localized effects in the nerve endoneurium is a current bottleneck in research and development (80). Emerging technologies, such as nanoparticle carriers and targeted peptides, show promise but are still in the early stages of development (81).

## 5 Future directions and new therapeutic perspectives

Overcoming the current treatment bottlenecks for DN requires technological innovation, in-depth basic research, and the strategic design of translational medicine. A core technological driver for the future will be the application of single-cell and spatial omics to map the immune-metabolic landscape of DN. Using single-cell RNA sequencing (scRNA-seq) and spatial transcriptomics, it will be possible to directly analyze nerve biopsy samples from DN patients, revealing specific immune and stromal cell subsets in the affected tissue, along with their unique metabolic and functional characteristics at unprecedented resolution.

Based on this, it will be crucial to use spatial omics technology to compare the characteristics of macrophage subsets in the perineurium and endoneurium, identifying the cells that provide barrier protection in the outer perineural layer and the pathogenic core subsets that invade the endoneurium to drive damage. This approach will form the foundation for stage- and site-specific precision interventions. Additionally, developing organoids or “neurosystems on a chip” that incorporate neurons, SCs, immune cells, and microvascular units will better simulate the complex interactions *in vivo*, providing platforms for high-throughput drug screening closer to physiological states.

In-depth exploration of key scientific questions is also crucial, particularly identifying the “switch” that drives immune responses from protective to destructive (82). Investigating the impact of the “gut-brain axis” holds great potential, aiming to elucidate how gut microbiota and their metabolites can modulate inflammation in peripheral nerves from a distance (83). Moreover, exploring the communication mechanisms between central and peripheral immune responses and revealing the interaction between spinal microglia activation and peripheral injury signals could help break the “central-peripheral” vicious cycle (84).

Moving toward precision therapeutic strategies requires constructing multidimensional intervention pathways based on the immune-metabolic network. First, targeting key immune-metabolic nodes through the development of selective NLRP3 inflammasome inhibitors, such as MCC950, can precisely block the critical transition from metabolic stress signals (e.g., ROS, lipids) to inflammatory signals (e.g., IL-1β). Second, reprogramming immune cell metabolism to restore energy homeostasis in immune cells under the “metabolic deadlock” or “metabolic paralysis” conditions in the diabetic environment using metabolic reprogramming drugs. The most forward-thinking strategy involves using AMPK agonists (e.g., metformin) or PPARγ agonists to guide macrophages from a pro-inflammatory M1 phenotype to a pro-repair M2 phenotype, thus restarting immune cell repair functions and overcoming the impasse of inflammation resolution failure. Finally, applying recombinant MANF protein and other endogenous modulators to restore neurotrophic support, combined with immune-metabolic regulators, and antidiabetic, antioxidant, or analgesic drugs in a multi-target combination treatment regimen, will be key to achieving optimal therapeutic efficacy.

## 6 Conclusion

The pathogenesis of DN is far more complex than previously understood. The paradigm shift from a simplistic view of “metabolic toxicity” to an integrated “immune-metabolic network” provides a fresh perspective on this persistent disease. In this network, hyperglycemia and lipotoxicity are no longer merely direct damaging factors for neurons; they also serve as fuel to ignite the immune system’s “inflammatory engine.” Immune cells, through their intrinsic metabolic overload and “metabolic paralysis,” convert systemic metabolic imbalance into persistent and refractory local nerve damage.

This process not only involves the dysregulation of immune cells but also includes the transformation of SCs from metabolic supporters to inflammation contributors. The severe impairment of the lactate shuttle mechanism, driven by this apparent “zero-sum game,” places axons in an inescapable state of “hunger and toxicity.” Nevertheless, it is crucial to recognize Schwann cell plasticity; depending on local microenvironmental conditions and the specific stage of the disease, these cells may still sustain certain residual metabolic support functions. This profound interaction ultimately triggers a cascade reaction mediated by the PANoptosome, forming a vicious cycle that drives the progression of DN.

Although we still face significant challenges in translating basic research into clinical applications—such as model disconnects, spatiotemporal heterogeneity, and drug delivery—the introduction of the immune-metabolic network theory has pointed us in the right direction. By utilizing cutting-edge technologies such as single-cell omics to precisely identify metabolic subpopulation differences between the perineurium and endoneurium, and by developing therapies targeting key nodes (such as NLRP3) or restarting immune-metabolic repair functions through metabolic reprogramming, we have reason to believe that, in the near future, we can break the current “symptomatic treatment” impasse in DN. Making the immune-metabolic network the core intervention target is undoubtedly the key path to this future.

## Data Availability

The original contributions presented in the study are included in the article/supplementary material. Further inquiries can be directed to the corresponding authors.
